# Cross-sectional multimedia audit reveals a multinational commercial milk formula industry circumventing the Philippine Milk Code with misinformation, manipulation, and cross-promotion campaigns

**DOI:** 10.3389/fnut.2023.1081499

**Published:** 2023-02-02

**Authors:** Donna Isabel S. Capili, Janice Datu-Sanguyo, Claire S. Mogol-Sales, Paul Zambrano, Tuan T. Nguyen, Jennifer Cashin, Roger Mathisen

**Affiliations:** ^1^Kalusugan ng Mag-Ina, Inc., Quezon City, Philippines; ^2^Alive & Thrive East Asia Pacific, FHI 360, Muntinlupa, Philippines; ^3^Breastfeeding Support Peer Mothers Group, Manila, Philippines; ^4^Alive & Thrive East Asia Pacific, FHI 360, Manila, Philippines; ^5^Alive & Thrive East Asia Pacific, FHI 360, Hanoi, Vietnam; ^6^Alive & Thrive East Asia Pacific, FHI 360, Washington, DC, United States

**Keywords:** breastfeeding, commercial milk formula, content analysis, cross-promotion, inappropriate marketing, media monitoring, Philippines, the Code

## Abstract

The Philippine Milk Code was enacted in 1986 to protect breastfeeding and reduce inappropriate marketing of breastmilk substitutes (BMS). The Philippine Milk Code is categorized as “substantially aligned” with the International Code of Marketing of Breast-milk Substitutes (“the Code”), but its provisions are assessed as relatively weak in prohibiting promotion to the general public. The extent to which violations of the Philippine Milk Code persist in traditional media platforms and in the digital space has not been systematically explored. This study employed a cross-sectional multimedia audit to examine the marketing and promotion of products under the scope of the Code, as well as those regulated by the Philippine Milk Code. Through a media monitoring conducted from March to September 2018, a total of 430 unique television (*n* = 32), printed (*n* = 87) and online (*n* = 311) promotional materials were identified. A coding tool was used to analyze the content, including the marketing elements used in the materials. Our findings show that commercial milk formula (CMF) for children ≥36 months old was the most promoted type of product (*n* = 251); and staging of events (*n* = 211), provision of special discounts or financial inducements (*n* = 115) and the use of taglines (*n* = 112) were the most used marketing elements. Promotion of CMF for children <36 months old was uncommon, which supports the conclusion that there is broad compliance with the Philippine Milk Code in terms of the types of products promoted. However, analysis of marketing elements reveals that the CMF industry circumvents the Philippine Milk Code through the use of false and misleading health and nutrition claims, emotionally manipulative language in promotional materials, and cross-promotion. The findings indicate gaps in enforcement and regulatory measures that require urgent attention.

## Graphical Abstract

Promotional materials for products under the scope of the Philippine Milk Code were examined using content analysis methods. The findings show evidence of circumventions of the Philippine Milk Code in modern and traditional media channels that exploit gaps in the legislation and its enforcement. 
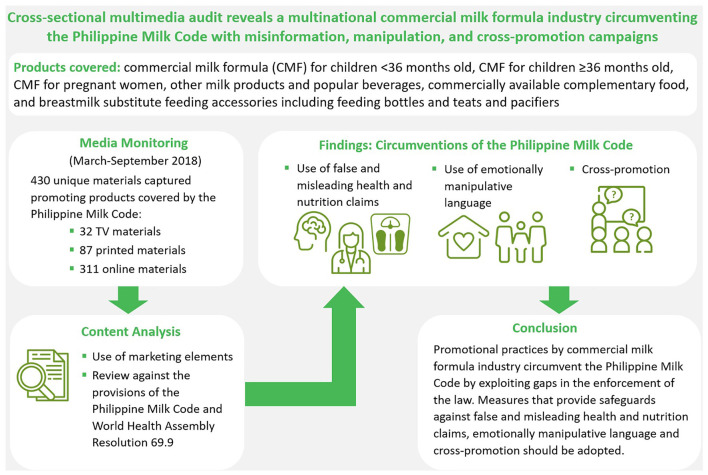

## Introduction

Breastfeeding is a life-saving intervention that averts 595,379 deaths among children 0–23 months old each year (1). Globally, the economic cost of not breastfeeding is estimated at USD 574 billion annually or 0.7% of the global gross national income (1, 2). Aggressive marketing and promotion of breastmilk substitutes (BMS), a type of commercial milk formula (CMF) marketed as partial or total replacements for breastmilk for children 0–36 months of age, has played a significant role in undermining breastfeeding (3). The World Health Assembly (WHA) introduced the International Code of Marketing of Breast-Milk Substitutes (“the Code”) to protect breastfeeding and reduce inappropriate marketing of BMS, artificial feeding accessories such as bottles and teats, and complementary foods when marketed as a replacement for breastmilk (4). However, inappropriate marketing of BMS has continued and violations of the Code persist across the world (5).

The Philippines was among the first countries to adopt a national legislation on the Code through Executive Order No. 51 s. 1986 (EO51), or the National Code of Marketing of Breastmilk Substitutes, Breastmilk Supplements and Related Products, more commonly known as the Philippine Milk Code. Revised Implementing Rules and Regulations (RIRR) were adopted in 2007 after a protracted legal battle against the formula milk industry (5). The Philippine Milk Code withstood several legal challenges (6, 7) and as of 2022, is still categorized as “substantially aligned” with the Code and is one of only 32 countries globally and one of the only two countries in Southeast Asia in this category (8). The Philippine Milk Code defines BMS as any food being marketed or otherwise presented “as a partial or total replacement of breastmilk” whether or not suitable for that purpose (9), thus not limiting its scope to any age group. Promotion of feeding accessories such as bottles and teats are also prohibited. In 2019, Republic Act (RA) No. 11148 or the First 1000 Days Act was enacted. Its provisions considered the WHA Resolution 69.9 recommendations to end the inappropriate promotion of food for infants and young children and defined BMS to include milk products “marketed for feeding infants and young children up to the age of three years” (10). This clarified that CMF for children younger than 36 months old are BMS. Products for children more than 36 months can be classified as BMS if they are marketed or otherwise presented as a partial or total replacement for breastmilk, in line with the scope of the Philippine Milk Code. The Philippine Milk Code has served as a strong legal basis for other supportive policies such as the Department of Health (DOH) Administrative Order 2007–0017, or the Guidelines on the Acceptance and Processing of Foreign and Local Donations during Emergency and Disaster Situations, which provides restrictions on donations of BMS during humanitarian emergencies (11). While the Philippine Milk Code’s provisions are strong in terms of scope, monitoring and enforcement, and in prohibiting promotion in healthcare facilities, they are relatively weak in prohibiting promotion to the general public and in prohibiting conflicts of interest in the health system (8).

In 2020, the size of the baby food market in the Philippines reached over USD 850 million, with sales of CMF products comprising 95% of the total market (12). In the last 15 years, minimal decline was recorded in the sales of formula products for children 0–12 months, but sales of formula milk for children 13–36 months and specialized formula significantly increased (13).

The prevalence of exclusive breastfeeding among all infants under 6 months of age is 57.9%, but by 6 months, only 35.1% of infants are still exclusively breastfeeding (14). Only 34.2% of infants continued to be breastfed up to 2 years (14). Every year, nearly 3 million avoidable cases of childhood diarrhea and pneumonia, 21,000 cases of Type 2 Diabetes in women, and 17,000 cases of childhood obesity are attributable to not breastfeeding according to recommendations in the Philippines and these translate to an annual economic loss of USD 16.3 million in health system costs and USD 2.3 billion due to cognitive losses associated with not breastfeeding (15).

Despite the strength of the Philippine Milk Code, violations persist in the country (16), including in the aftermath of humanitarian emergencies that occur with regularity. A sharp rise in reported violations in the Philippines during the early phase of the COVID-19 pandemic were among those highlighted in a recent multi-country study (17). The extent to which violations of the Philippine Milk Code persist in more traditional media platforms and in the digital space has not been systematically explored.

This study was conducted to examine the marketing and promotion of products under the scope of the Code, as well as the content of marketing and promotional materials regulated by the Philippine Milk Code. The findings support public health practitioners and others to identify and recommend appropriate policy actions to strengthen the implementation of the Philippine Milk Code, its implementing rules and regulations, and other related policies. The findings also contribute to the global evidence-base of persistent violations of the Code since its adoption and can be used to inform agenda setting and resolutions of the WHA.

## Materials and methods

This is a cross-sectional study to examine data collected from a multimedia monitoring of promotional activities for products covered by the Philippine Milk Code from March to September 2018.

### Products covered

The multimedia monitoring covered promotional materials for products under the scope of the Philippine Milk Code, including “infant formula; other milk products, foods and beverages, including bottled complementary foods, when marketed or otherwise represented to be suitable, with or without modification, for use as a partial or total replacement of breastmilk; feeding bottles and teats” (9). These include promotional materials for the following products:

• Breastmilk substitutes as defined in RA 11148, which are milk products marketed for children up to 36 months old (<36 months) (10). These products include CMF for children 0–5 months old, CMF for children 6–11 months old and CMF for children 12–35 months old.
• Other milk products, foods and beverages including CMF for children aged 36 months old or older (≥36 months), CMF for pregnant women (CMF-PW), milk products (in liquid or powder form) and other popular beverages (e.g., chocolate and malt drinks) marketed with no specific age range indicated, and commercially available complementary food (CACF).
• Breastmilk substitute (BMS) feeding accessories including feeding bottles and teats, and pacifiers.

### Setting

The Philippines is a lower middle-income country (18) in Southeast Asia with gross national product (GNP) worth USD 394.1 billion in 2021 (19). The country’s economy is driven by incomes from the services, industry, and agriculture, forestry and fisheries sectors (20) and is supported by strong labor market and consumer demand (21). In 2021, its population is estimated at 111 million (22). In 2020, 1.5 million live births were registered in the country (23). Rates of neonatal, infant and under-5 mortality rates were estimated at 12.6, 21.0, and 26.4 per one thousand live births, respectively (24). Functional literacy rate among Filipinos 10 to 64 years old was at 91.6% in 2019 (25). About 96.0% of Filipinos watch television (TV); 73.9 and 63.6% surf the internet for social media and for email or research works, respectively; and 63.3 and 73.2% read newspapers and magazines, respectively (26). About 130 languages are used in the Philippines (27) but Filipino is officially declared as the national language (28). English, which is widely spoken, is also considered as an official language in the country (28) and is commonly used as medium of instruction and business language.

### Data collection

The identification of marketing materials included in the study was guided by the process employed in a media audit by Vinje et al. which covered promotion of products under the scope of the Code in Cambodia, Indonesia, Myanmar, Thailand, and Vietnam (29). A local, independent firm (Media Meter) was engaged to perform systematic monitoring of TV, print, and online media materials. A set of keywords was used to guide the capture of materials (Supplementary Table 1). The keywords included manufacturer/company, brand, and product names as well as general keywords that included “malnutrition” and type of food, bottles, and teats (e.g., infant formula, follow-up formula, growing-up milk).

Data collection took place from March to September 2018. Monitoring of TV, which covered major TV networks and identified cable channels was limited to “primetime” (6:00 to 9:00 am and 6:00 to 9:00 pm). For printed materials, monitoring covered publications in print including major newspapers, tabloids, and magazines. The monitoring of online materials covered online news and magazine sites, blog sites and other websites. Due to the limitations in the monitoring firm’s capabilities, the monitoring did not include e-commerce sites or ad placements by online advertising platforms that display brief advertisements, service offerings, product listings, or videos to web users. Ad placements on social media platforms and mobile apps were also not included. The monitoring attempted to capture related social media posts (user generated content) from Facebook, Instagram, and Twitter but restrictions due to privacy policies limited the collection of content on these platforms. Therefore, social media content was not included in this study.

Materials captured from TV (video clips) and printed publications (scanned copies) were digitally archived and stored for content accessibility. For online content, the specific uniform resource locators (URL) where the captured content was found were recorded. Digital archiving of internet-based material was conducted. However, some links were found to be inactive or have errors in content (broken links) during follow-up.

All the materials were reviewed and manually coded by researchers who are fluent in English and Filipino, the two languages commonly used in media materials in the Philippines. For materials written in other languages, Google Translate was utilized to translate the materials to English.

### Data analysis

Conceptual analysis of materials was done using a coding tool developed and adapted from Zhao et al. (30) and Berry and Gribble (31), which are both based on content analysis methods. The coding tool included the following categories:

1. General information–documents product details: manufacturer or company name, brand or product name, and type of product.
2. Type of material–identifies if the material is a regular advertisement (TV commercial for TV, ad placement for print), news/feature article for print and online, or news/lifestyle program for TV, user generated content-blogs (for online), advertorial.
3. Marketing information or reference–information included in the materials for accessing the product or information about the product: retail outlet location, hotline number, and online access details such as website, email address and social media page.
4. Marketing elements–identifies the marketing techniques used in the material: special price or discount, voucher, free product sample, contest, event, corporate social responsibility or advocacy, availability for online purchase, free delivery, use of logo or tagline.
5. Emotional appeal–documents strategies used to engage feelings of the viewer: use of imagery such as celebrities, mascots, character representation (e.g., happy family), fictional or cartoon characters.
6. Rational appeal–includes strategies to engage reasoning of the viewer: health claims, nutrition claims, quality assurance claims, scientific recommendations, importation claims and promotional offers.

To determine potential violations and inappropriate marketing and promotion of products, the content of materials was analyzed against the provisions of the Philippine Milk Code and its RIRR (Table 1) and with WHA Resolution 69.9 recommendations. For the materials discussing events, the content was analyzed for the promotional elements used during the activity (as described in the materials), such as the presence of celebrities, distribution of free samples, discounts for participants and other promotional offers during the event.

**TABLE 1:** Key provisions of the 2006 revised implementing rules and regulations of the Philippine Milk Code.

Rule 5 – Advertising, promotion, marketing, and sponsorships
**Section 13. “Total Effect.”** Promotion of products within the scope of this Code must be objective and should not equate or make the product appear to be as good or equal to breastmilk or breastfeeding in the advertising concept. It must not in any case undermine breastmilk or breastfeeding. The “total effect” should not directly or indirectly suggest that buying their product would produce better individuals, or resulting in greater love, intelligence, ability, harmony or in any manner bring better health to the baby or other such exaggerated and unsubstantiated claim.
**Section 15. Content of Materials.** The following shall not be included in advertising, promotional and marketing materials: a. Texts, pictures, illustrations or information which discourage or tend to undermine the benefits or superiority of breastfeeding or which idealize the use of breastmilk substitutes and milk supplements. In this connection, no pictures of babies and children together with their mothers, fathers, siblings, grandparents, other relatives or caregivers (or *yayas*) shall be used in any advertisements for infant formula and breastmilk supplements; b. The term “humanized,” “maternalized,” “close to mother’s milk” or similar words in describing breastmilk substitutes or milk supplements; c. Pictures or texts that idealize the use of infant and milk formula.
**Rule 6 – Prohibited acts**
**Section 16.** All health and nutrition claims of products within the scope of the Code are absolutely prohibited. For this purpose, any phrase or words that connote to increase emotional, intellectual abilities of the infant and young child and other like phrases shall not be allowed.
**Section 17.** False or misleading information or claims of products within the scope of the Code are prohibited.
**Section 19.** Manufacturers, distributors and marketing firms or representatives of products within the scope of this Code are prohibited from donating or giving directly or indirectly, samples and supplies to any members of the general public, to hospitals, and other health facilities, including their personnel and members of their families.
**Section 20.** Manufacturers, distributors and marketing firms or their representatives of products within the scope of this Code are prohibited from using the health workers and the healthcare system in the dissemination, distribution and promotion of products within the scope of the Code.
**Section 21.** Gifts of any sort from milk companies/manufacturers, distributors, and representatives of products within the scope of this Code, with or without company name or logo or product or brand name shall not be given to any member of the general public, to hospitals and other health facilities, including their personnel and members of their families.
**Section 22.** No manufacturer, distributor or representatives of products covered by the Code shall be allowed to conduct or be involved in any activity on breastfeeding promotion, education and production of information, education and communication (IEC) materials on breastfeeding, holding of or participating as speakers in classes or seminars for women and children activities and to avoid the use of these venues to market their brands or company names.
**Section 23.** There shall be no point-of-sale advertising, giving of samples or any promotion devices to induce sales directly to the consumers at the retail level, such as special displays, discount coupons, premiums, rebates, special sales, bonus and tie-in sales, loss-leaders, prices or gifts for the products within the scope of this Code.

## Results

**FIGURE 1:**
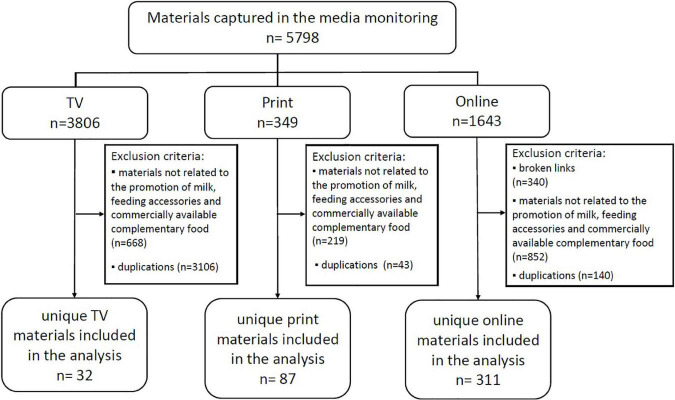
Methods process flow for the identification of unique TV, printed, and online materials for detailed analysis.

A total of 5,798 materials were captured within the monitoring period. Materials screened included 3,806 TV materials, 349 print materials, and 1,643 pieces of online content. Figure 1 illustrates the screening of materials for inclusion in the analysis. Overall, 430 unique marketing materials were identified for analysis. Most captured materials were in English, Filipino, or a combination of both. Only five, all of which were online materials, were written in another language (Cebuano).

### Promotion by type of product

**FIGURE 2:**
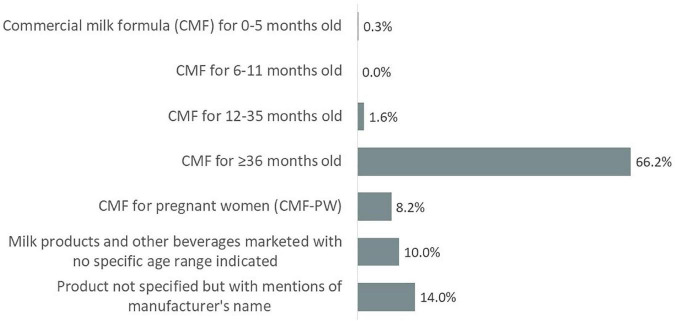
Distribution of CMF and milk products and other beverages marketed with no specific age range indicated promotion in unique materials (*n* = 379).

Of the 430 unique materials captured across multimedia, 379 promoted CMF and milk products and other beverages with no specific age range indicated. Of the 379 materials, CMF for children ≥36 months old was the most commonly promoted type of product (66.2%) (Figure 2). Fourteen percent mentioned only the manufacturer’s name and did not mention any specific product being promoted while 10.0% promoted other milk products and beverages marketed with no specific age range indicated. Promotion of CMF-PW were found in 8.2% of these materials while 1.9% included promotion for CMF for children <36 months.

For CACF promotion, 39 unique materials were captured, all of which promoted Nestle products.

For BMS feeding accessories, 19 unique materials were found promoting 12 brands of feeding bottles and teats and 3 brands of pacifier. Two materials mentioned only the names of bottles and teats manufacturers and two other materials promoted the use of pacifiers but did not mention a particular brand.

### Mass media coverage

Of the 430 unique materials captured for monitoring, online media had the highest share of unique content (72.3%), followed by print materials (20.2%) and TV (7.4%). Table 2 presents the types of products promoted across media channels monitored.

**TABLE 2:** Number of unique materials by the type of product across multimedia platforms monitored.

Type of product	TV (*n* = 32)	Print (*n* = 87)	Online (*n* = 311)	Total (*n* = 430)
**Commercial milk formula (CMF) and milk products and other beverages marketed with no specific age range indicated**	**31**	**78**	**270**	**379**
CMF for 0–5 months old	0	0	1	1
CMF for 6–11 months old	0	0	0	0
CMF for 12–35 months old	4	1	1	6
CMF for ≥36 months old	26	50	175	251
CMF for pregnant women (CMF-PW)	0	11	20	31
Milk products and other beverages marketed with no specific age range indicated	0	11	27	38
Product not specified but with mentions of CMF/milk product manufacturer’s name	1	11	41	53
**Commercially available complementary food (CACF)**	**1**	**8**	**30**	**39**
Cerelac (Nestle)	1	8	29	38
Gerber (Nestle)	0	0	1	1
**Breastmilk substitute (BMS) feeding accessories**	**0**	**1**	**18**	**19**
Bottles and teats	0	1	14	15
Pacifiers	0	0	5	5
Product not specified but mentions BMS feeding accessory manufacturer’s name	0	0	2	2

Words in bold indicate product category. Values in bold indicate number of unique materials per product category.

#### Television

The 32 unique TV materials captured in the monitoring aired on seven different TV channels and included 26 TV commercials (TVCs) and six video clips from news and lifestyle TV programs. CMF for children ≥36 months was the most commonly promoted product on TV (26 unique materials). Four materials promoted CMF for children 12–35 months old. There was one material which promoted a CACF product manufactured by Nestle. No TV material for BMS feeding accessories was captured in the monitoring. Of the 26 TVCs, promotions for Nestle accounted for 73% (19/26), followed by Abbott at 15% (4/26) and Reckitt at 12% (3/26). The other six materials which were presented as segments of news and lifestyle programs promoted Nestle products.

#### Print

Of the 87 captured unique print materials, 15 (17%) were ad placements and 72 (83%) were presented as either news reports or feature articles. Eleven of the 15 ad placements were advertisements of supermarket chains operating under SM Retail, Inc. (Pasay City, Philippines), [SM Supermarket (*n* = 4), SM Hypermarket (*n* = 4) and Savemore (*n* = 3)], which featured CMF and milk products and other beverages marketed with no specific age range indicated along with other products that they offer (an example of this supermarket advertisement is shown in Supplementary Figure 1). Three ad placements were found to promote events co-sponsored by milk manufacturers. CMF for children ≥36 months was the most promoted type of product, which was found in 57% (50/87) of the unique printed materials. Eight printed materials promoted a CACF brand from Nestle and one material promoted a brand of bottles and teats. Nestle had the most promotions in print (87% or 76/87).

#### Online

A total of 311 unique online promotional materials were captured in the media monitoring. Most of the products promoted were brand variants of CMF for children ≥36 months, which were found in 58.5% (182/311) of the unique online materials analyzed. Promotion for Nestle was found in 82.3% (256/311) of the materials and promotion for Nestle’s brands of CMF for children ≥36 months was collectively present in 49.8% (155/311) of the materials. Promotion of milk products and other beverages marketed with no specific age range indicated was seen in 10.9% (34/311) of the materials.

Twenty-nine of the 30 CACF online materials captured promoted Cerelac, a brand from Nestle. One material promoted another CACF brand from Nestle but it was directed to an audience overseas. All 20 online materials captured for CMF-PW were promoting a single brand from Nestle. There were also 18 unique materials for BMS feeding accessories which promoted 14 unique products. By type of material, 210 were user-generated content in the form of blogs and 97 were online news and lifestyle feature articles. Four were advertorials or content disclosed as being paid or sponsored content.

Overall, promotion on primetime TV was the most extensively utilized through airing of TVCs. While there were only 26 unique TVCs captured, they were broadcast 3,132 times during the period covered, showing the use of repetition as a marketing technique. The majority of TVCs were aired on ABS-CBN Channel 2 (*n* = 1,231) and GMA 7 (*n* = 1,859), two major TV channels operating on free TV at the time of the media monitoring. While each TVC ran for 15–60 s, collectively all TVCs captured in the monitoring had a total running time of 78,390 s. The high number of unique materials captured from various websites and blog sites likewise show widespread promotion online.

### Promotional content

#### Marketing information

Materials reviewed commonly contained guidance on how to access online information about the products. Among the 32 TV materials, two included the product websites, another two gave keywords for an online search to get more information about the product, and three others provided links to related social media pages. For print, 11 advertisements of supermarket chains operating under SM Retail, Inc. (Pasay City, Philippines), which featured CMF products and other milk and beverages included links to SM Markets website. The website presented listings of promotions offered and available goods for sale. However, there were no specific manufacturer nor products’ direct marketing information shared in these supermarket ads. Seventeen printed news and feature articles included the company or product website or social media page, or websites of e-commerce platforms where the products can be purchased. For online, about 37.9% (118/311) of materials reviewed provided links to websites for accessing more information about products. These included 63 links to Lazada and Shopee websites, two of the largest e-commerce platforms in the country where consumers can purchase the products.

Two TV and three online materials provided phone numbers to call for product information. Names of physical stores where products retailed were included in eight online materials while two TVCs mentioned availability of products in “suking tindahan,” or neighborhood stores.

#### Marketing elements

The materials reviewed showed a variety of promotional tactics aimed at engaging consumers. A strategy could include more than one marketing or promotional element. Table 3 lists the frequency of these marketing elements across multimedia as seen in the review.

**TABLE 3:** The number of used marketing elements in unique materials across multimedia platforms monitored.

Marketing element	TV (*n* = 32)	Print (*n* = 87)	Online (*n* = 311)	Total (*n* = 430)
Event	6	45	160	211
Special price/discount/financial inducement	2	25	88	115
Tagline	31	11	70	112
Logo/product shot	26	14	28	68
Available for online purchase	0	5	62	67
Others (Contests, free product sample, free item, interactive game, free delivery, corporate social responsibility activity)	1	7	36	44

##### Events

Staging events was the most frequent (*n* = 211) marketing element used across multimedia. A series of events with a single theme to promote a particular brand were reported in a number of print feature articles and online postings (feature articles or blogs) over the review period. An example is the series of events for Nestle’s Promil Four i-Shine Talent Camp which promoted a brand of CMF for children ≥36 months. Our monitoring captured a total of 15 feature articles from printed publications, 31 online postings, and 5 TV materials about the i-Shine Talent Camp across 5 months (examples of these materials are shown in Supplementary Figure 2). Other examples were the (a) Grow Happy Nation events for a Nestle brand of CMF for children ≥36 months, which offered interactive activities for parents and children (featured in 4 print and 23 online materials released in 4 different months); (b) parenting events promoting another Nestle brand of CMF for children ≥36 months (presented in 4 print and 19 online materials captured in five consecutive months); and (c) Let’s Eat Bulilit campaign activities to promote a Nestle CACF product including participation by parents and their kids (found in 3 print and 15 online materials published within five consecutive months). Some materials that featured events also featured celebrities (4 TV materials, 23 print materials, and 84 online materials). There were materials promoting events co-sponsored or supported by milk manufacturers, such as a commemorative activity in a local government unit, a micro entrepreneur convention, and a raffle promo of a supermarket chain.

##### Special pricing/discounts/financial inducements

Offers of special pricing or discounts were also commonly included in materials reviewed. Among the TVCs, there were two materials promoting brands of CMF for children ≥36 months that advertised a suggested retail price for product size variations. For print, 12 ads as well as 13 news and feature articles included promotional discounted prices. About 92% (23/25) of these promoted CMF for children ≥36 months. For online, 88 materials featured special prices or discounts. About 78% (69/88) of these were for products classified as CMF for children ≥36 months while 14% (12/88) mentioned only the milk manufacturer/company names and did not specify products for which the special prices or discounts apply. Five online materials advertised discounts for BMS feeding accessories. One blog posted about a special deal for a brand of CMF for children 0–5 months and one CACF brand, but it was directed to an audience overseas. Around 68% (60/88) of the online materials which included special prices or discounts were part of promotional content of e-commerce platforms Shopee and Lazada. Information on the availability of products for online purchase (e.g., availability in Shopee or Lazada) was also identified in 70% (62/88) of online materials.

##### Logos and taglines

In all the captured unique TVCs (*n* = 26), product shots and logos were prominently visible. For print materials, 14 of the 15 ad placements utilized product shots or logos. Among the 311 online materials, logos were visible in 28 materials. Company or brand logos could be part of the promotional materials for an event, a stock image used by the content producer or writer, or photograph of the actual product. News and feature articles from online platforms were largely written in text, whereas user generated content by bloggers usually featured their own pictures or stock images. Taglines were used in 31 TV, 11 print, and 70 online materials. Table 4 lists the taglines of products found in the materials.

**TABLE 4:** List of products and taglines in promotional materials captured in media monitoring.

Manufacturer/company	Brand/product	Tagline
**Abbott**	PediaSure Plus (CMF for ≥36 months)	• See visible growth in just 8 weeks
	New Similac GainSchool (CMF for ≥36 months)	• Strong immunity for faster learning
**FrieslandCampina/Alaska Milk Corporation**	Alaska Powdered Milk Drink (CMF for ≥36 months)	• *Wala pa ring tatalo sa Alaska* (still nothing beats Alaska)
**Reckitt/Mead Johnson**	Enfagrow A+ Four (CMF for ≥36 months)	• Help nourish your child’s greatness right from the very start • For the IQ and EQ advantage
	Lactum 3+ (CMF for ≥36 months)	• *Ibang Level BIBO Kid* (Higher Level, ACTIVE Kid) • #FunMealTimeTransformation
**Nestle**	Bear Brand Fortified Powdered Milk (CMF for ≥36 months)	• *Para ang Tibay Always Present* (Endurance Always Present) • *Mag-Bear Brand Araw-Araw* (Bear Brand Everyday) • *Magcocotumbas sa Tibay!* (“Magcocotumbas” in Endurance) • *Pinipili ang Napatunayan, Bear Brand Araw-araw* (choosing what was proven, Bear Brand everyday)
	Nankid Optipro Four (CMF for ≥36 months)	• Help reshape your child’s future today/#ReShapeTheFuture
	Nestokid 4/Nestogrow4 (CMF for ≥36 months)	• Grow happy Shalalala
	Nido Advanced Protectus 3+ (CMF for ≥36 months)	• Love that protects
	Nido Fortigrow (CMF for ≥36 months)	• Your love. Their future.
	Cerelac (CACF)	• *Let’s Eat Bulilit!* (let’s eat, little one!)
	Bonakid Pre-School 3+ (Nestle) (CMF for ≥36 months)	• *Pag 3-Pataas, Mag-Bonakid Pre-school 3*+*; Batang Matatag, Batang may Laban* (For 3 years old and above, Bonakid Pre-school 3+; strong child, child who has fighting chance)
	Promil Four (Nestle) (CMF for ≥36 months)	• Nurture the gift
	Promil Gold Four (Nestle) (CMF for ≥36 months)	• Advance today’s gift

#### Emotional appeal

Among the TVCs, the most commonly employed strategy was having celebrity endorsers (*n* = 7) and brand jingles (*n* = 7). Images of a happy family were shown in four materials. Two fictional or cartoon characters were also used among the TVCs–one was a bear for promoting one of Nestle’s CACF product, and the depiction of the “Batang May Laban (Strong Kid)” for a Nestle’s brand of CMF for children ≥36 months. A mascot, the Nestle blue bear, was also featured in one TVC.

About 37% (32/87) and 32.1% (100/311) of print and online materials, respectively, featured celebrity endorsers. The same celebrities were commonly featured in multiple materials on multiple channels. For example, a celebrity who was a new father was featured in 22 marketing exposures (1 TVC, 4 print and 17 online materials) for a Nestle’s CACF product. In nine of these materials, the father was shown alongside his baby. One material described that the father was chosen to be the face of the brand because “he is a certified millennial parent aspiring to be the best dad ever.”

There were also those which featured more than one celebrity in a single promotional material, such as a blog entry about a parenting event for the promotion of one of Nestle’s brand of CMF for children ≥36 months, which featured five celebrities who are all mothers to pre-school children. There was also a pre-school age brand ambassador for one of Nestle’s brand of CMF for children ≥36 months, who is the child of a celebrity couple and has significant social media following. For one of Nestle’s brands of CMF for children ≥36 months, a famous composer and musician was engaged for their campaign about nurturing children’s talents.

The use of mascots (or brand character) was among the least observed marketing tactics in this review. Only six materials (1 TVC and 5 online materials) employed the use of a brand character, which were all for the promotion of one Nestle CACF brand. The brand character was shown in the materials from pictures of the product packaging or in photographed appearance of the mascot in events. Pictures of family members or children with or gathered around the products were also observed in 49 materials, which were mostly pictures of bloggers themselves with their children and other participants of the events they attended.

#### Rational appeal

All the TVCs reviewed, including promotion of CMF for children ≥36 months (*n* = 17) and milk products and other beverages marketed with no specific age range indicated (*n* = 4), contained health and nutrition claims. However, the materials for the four milk products and one CACF marketed for toddlers below 36 months only contained information on its suggested retail price, packaging sizes, flavor variants, and retail outlets.

Among the print and online materials, not all included health or nutrition claims. For example, the supermarket advertisements among the printed materials only featured product shots and information on prices. Articles about sales promotions, discounts, co-sponsorship of events, in print and online, also did not include health or nutrition claims. But health and/or nutrition claims were found in 32.2% (128/398) of the print and online materials (60 for CMF for children ≥36 months, 20 for milk products and other beverages marketed with no specific age range indicated, 25 for CMF-PW, 17 for CACF, 6 for BMS feeding accessories). Table 5 lists examples of health and nutrition claims for CMF for children ≥36 months, milk products and other beverages marketed with no specific age range indicated and CACF in TV, print and online materials. Note that as was the case with the TV materials, there were no materials claiming health and nutrition benefits for milk products marketed for children <36 months. Table 5 also lists health benefit claims related to BMS feeding accessories, as identified in print and online materials. And although there were only six materials captured with claimed benefits for BMS feeding accessories, the materials presented different products across eight brands.

**TABLE 5:** Examples of product benefit claims in unique promotional materials.

Health claims in materials promoting CMF for children ≥36 months and CACF							
**Functional processes**		**Growth and development effects**			**Physiological process or outcome**		**Protection against disease**
• Easier digestion • Facilitates energy release from food • Contributes to normal function of the immune system • Helps develop beneficial intestinal microflora • Aids in better calcium absorption • Fast absorption of nutrients		• Supports your child’s growing needs • Supports overall growth and development • Helps strengthen immunity • Faster growth and development • Makes sure bone growth is normal and consistent • Support your child’s cognitive and emotional development • Helpful for growth and mental development • Important building block for brain and eye development • Support mental and physical development • Boosts not just intelligence but all 8 signs of brain development • Helps to support age-appropriate weight gain and development • Helps strengthen the immune system • Supports brain and eye development			• Provides gut comfort • Help kids reach ideal height and weight • Supports a healthy digestive system • Helps perform well in school • Helps improve digestive health • Strengthens tummies to provide better digestion		• Helps protect against infections • Scientifically proven to help support your child’s respiratory defenses • To help reduce the risk of obesity of your kid later in life • Helps prevent micronutrient deficiency
**Nutrition claims in materials promoting CMF for children ≥36 months, milk products and other beverages marketed with no specific age range indicated and CACF**							
**Protein**	**Mineral**		**Carbohydrates**	**Fat**		**Biologically active ingredients**	**Others**
• High quality protein • High quality, lower quantity protein • Triple protein complex • Added whey protein	• More bioavailable Calcium • 2.5× more Vitamin D • Fortified with iron and zinc		Human milk oligosaccharides	• Lecithin • Milk fat globule membrane • DHA • DHA and ARA		• Naturally rich in antioxidants • Bifidus BL • L. comfortis • Probiotics/prebiotics • *L. rhamnosus*	• No sugars, no additives • Nutrient dense with no preservatives • Contains 1/3 of a child’s daily energy requirements • Nutrissentials–combination of unique and important nutrients • No added sucrose
**Benefit claims in materials promoting breastmilk substitute feeding accessories**							
• Promotes natural latch on • Minimizes nipple confusion • Lessens milk backflow and therefore mid-ear complications • Material that is closest to human skin • Has no open pores to harbor bacteria • No drip air vent helps prevent colic • Allows natural tongue movement • Anti-colic • Minimizes swallowed air and prevents gas					• Adapts to child’s palate for natural drinking experience • Approved by the oral health foundation • Promotes correct mouth development • Ergonomic and encourages correct neck positioning • BPA free • Allows easy grip • Non-toxic • Heat resistant up to 180 degrees Celsius • Protects against germs–nipple pops back into protective bubble when it falls		

For the materials promoting CMF for children 12–36 months, four provided information on the introduction of variants’ new package sizes and corresponding suggested retail price while another ad announced a new formulation. None used any other marketing element nor mentioned health or nutritional claims.

### Cross-promotion

Marketing elements used in the promotional materials were further examined to detect cross-promotion (Supplementary Table 2). There is cross-promotion when promotional elements of a CACF or beverage appear very similar to those of the company’s range of CMF products for <36 months, effectively promoting the latter.

**FIGURE 3:**
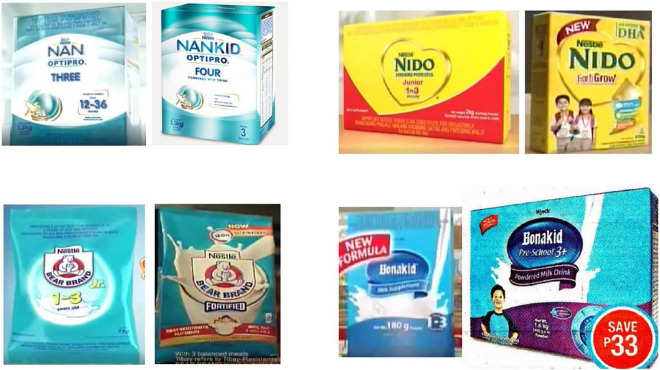
Images of eight products from the reviewed materials showing cross-promotion through similar packaging, branding, and labeling of CMF for children less than 36 months old **(left)** and CMF for children 36 months or older **(right)**.

Figure 3 presents images of eight milk products from the materials analyzed. The products on the left were marketed as CMF for children 12–35 months, and the respective products on the right are CMF for children ≥36 produced by the same manufacturer. It is noticeable how similar the packaging, branding, and labeling of the specific CMF product for 12–36 months to the corresponding CMF for children ≥36 months.

There were only five promotional materials for CMF for children 12–35 months identified across the multimedia platforms monitored. The four CMF for children ≥36 months included in Figure 3 had a total of 95 product mentions in unique promotional contents across multimedia platforms monitored. Various marketing techniques were utilized in the promotion of the materials, including the use of health and nutrition claims. Further, 12.3% of all promotional materials carried only the name of the milk manufacturer or company, which indirectly promoted all the products the companies manufacture, including CMF for children <36 months. The media monitoring also documented promotions of CMF-PW manufactured by Nestle. Marketing of CMF-PW presents opportunities for cross-promoting other types of CMF, including CMF for children <36 months.

## Discussion

This study was conducted to examine the marketing and promotion of products under the scope of the Code and to analyze the content of promotional materials regulated by the Philippine Milk Code through a media monitoring. The distribution of promotional materials captured in the media monitoring, in which only one of 430 unique materials was found to promote CMF for children 0–6 months, is consistent with a global trend in CMF market growth which is increasingly driven by sales of products marketed for children 12–36 months (32, 33). This finding may initially be considered as a reflection of this trend rather than as evidence of local enforcement of the Philippine Milk Code. However, the relative absence of promotion for CMF for children <36 months compared with CMF for children ≥36 months in this study supports the conclusion that there is broad compliance with the Philippine Milk Code in terms of the types of products promoted.

However, analysis of marketing elements of materials reveals how companies circumvent the Philippine Milk Code through false and misleading health and nutrition claims and the use of emotionally manipulative strategies in promotional materials. The Philippine Milk Code prohibits the use of false or misleading information or claims for products within its scope (34). The use of health and nutrition claims was not seen in materials promoting CMF for children <36 months but was extensively used in promoting CMF for children ≥36 months. The use of emotional and aspirational approaches in product promotion, a known practice by formula milk companies (35), was also observed in this review. Language that evokes emotional connection to the brand, such as the use of taglines about love, happiness and nurturing children’s gifts and future, were used in the materials promoting CMF for children ≥36 months. Endorsement of products by celebrities, or the TVCs depicting scenes that mirror ideal life situations and casting actors to whom the target market can easily relate with, evoke consumers’ aspirations of a good life or better future for their children and family. The use of false or misleading information or claims and of emotionally manipulative strategies are established exploitative marketing approaches (35) even if the latter is not explicitly prohibited by the Philippine Milk Code. Coupled with the common use of brand-based marketing, the net effect can also be considered cross-promotion from CMF for children ≥36 months to CMF for children <36 months. CMF for children ≥36 months was the most promoted in all media and its constant and ubiquitous promotion normalizes the use of CMF and undermines breastfeeding. This is evidence of a gap that requires urgent attention by both implementers of the Philippine Milk Code and policymakers at the level of the DOH and the legislative bodies.

Despite the Philippine Milk Code having been assessed as strong in terms of required and prohibited content in labeling and promotional material for CMF and related products (8), we found that TVCs and print ads for CMF for children 12–36 months contained no statements on the benefits and superiority of breastfeeding as required by Section 5 of the Philippine Milk Code. We also observed that TVC voice-over of statements such as “Use of milk supplements must only be upon the advice of a health professional,” and “Unnecessary and improper use may be dangerous to the child’s health” were sped up to fit into TVCs’ running time, severely compromising viewer comprehension of the message.

Further, for CMF for children ≥36 months, disclosure statements provided merely read “it is not suitable for infant feeding and is not a BMS.” The use of this disclosure statement is unclear as “infant” is commonly defined as a child within the age range of 0–12 months, and some might take to mean that the product is also suitable for feeding children between 12 and 35 months old. For CACF, disclosure statements read “It is not a BMS. Infants 6 months onward should be given fresh, indigenous, and natural foods in combination with breastfeeding.” There is generally no supportive messaging on recommended infant and young child feeding as these are not explicitly required by the Philippine Milk Code.

Findings of a study commissioned by the National Nutrition Council indicate that Cerelac, the brand promoted in 38 of the 39 CACF materials captured in the monitoring, is commonly fed to Filipino infants with some being introduced to the product even before they reach 6 months of age Gordoncillo et al. (36). Our findings show the use of health and nutrition claims and the use of pictures of a father together with a baby in the promotion of this product. The WHA 69.9 guidance document has pointed out that inappropriate marketing of CACF can mislead and confuse caregivers about the products’ nutrition and health-related qualities and about their age appropriateness and safe use (37). It can also lead caregivers to think that family foods are inadequate and create product dependency (37).

This study also documented promotions of BMS feeding accessories and the use of various benefit claims which idealize the use of these products. Such promotions normalize bottle feeding and undermine breastfeeding.

The Philippine Milk Code prohibits promotion through financial inducements at retail level. While evidence of such inducements was found for the promotion of CMF for children ≥36 months, coverage of these products by the Philippine Milk Code is still subject to case-by-case deliberation and this gap can be capitalized for inappropriate promotion.

Company hosting of or publicized participation in events is a common strategy seen in this analysis. Holding of events to promote products is closely linked to experiential marketing, a marketing technique which encourages consumer interaction and product trial (38). These events create opportunities for other forms of promotion that violate the Philippine Milk Code’s prohibitions on the giving, directly or indirectly, of samples and supplies of products within its scope, or gifts of any sort to any member of the public (9); and prohibitions against distribution by manufacturers or distribution of any gifts or articles or utensils that may promote CMF for children <36 months to pregnant women or mothers of infants (9). The Philippine Milk Code, however, does not explicitly prohibit contact with mothers and caregivers, and this is an acknowledged gap in the legislation (8). Hosting of events by CMF companies targeting participation of health workers is well documented (35) but the use of experiential marketing involving direct participation of parents, caregivers and children is a trend that should be closely monitored for possible use as venues for inappropriate marketing of products covered by the Code. We also found promotional materials documenting activities that may not have been directly initiated by CMF manufacturers but are nonetheless prohibited by the Philippine Milk Code. Examples included a supermarket chain announcing special prices for products within the scope of the Philippine Milk Code, a private company’s event with co-sponsorship from a milk manufacturer during which the latter distributed co-branded “giveaways,” and a local government unit activity that was co-sponsored by a milk manufacturer. These would be considered non-advertising promotion activities (37). It is not possible to ascertain from the gathered materials whether any of the promotional material used in the documented activities was evaluated by the relevant authorities.

We found evidence of circumventions of the Philippine Milk Code that proliferate in the digital space. Blog posts of individuals or celebrities, which include photographs of children with the product, can be considered violations of the Philippine Milk Code which prohibits the use of images (such as infants/children together with mothers) that may discourage or undermine breastfeeding or idealize the use of CMF and bottle-feeding.

Other possible violations of the Philippine Milk Code overlap in digital platforms. For example, bloggers covering the same events that involve CMF manufacturers used identical wording in their posts. At least eight bloggers posted about a Nestle event in May 2018, with the same title and text for all their posts (including unsubstantiated health and nutrition claims) suggesting that the postings were coordinated. This is in line with recent documentation of CMF companies engaging social media influencers including “mommy bloggers” to promote their products online, with some utilizing digital marketing techniques to reach pregnant women and mothers to influence their infant feeding decisions (39, 40). Only two of the 117 blog sites captured in the monitoring had disclosure statements about sponsored content. This type of Philippine Milk Code circumvention is not only restricted to bloggers but also to news publication outlets with both print and online formats. While many of the news and feature articles online and in print appear to be “advertorials” or sponsored content, only four articles gave such disclosures. This is a clear enforcement gap that is not currently addressed by the Philippine Milk Code.

This study is one of few to explore the content of marketing and promotional materials for multiple CMF, CACF, and BMS feeding accessory products across predominant media channels in the Philippines. However, our study had some limitations including the fact that this review which was limited to retrospectively examining generated media captures from March to September 2018, likely underestimated the extent of marketing and promotion of products covered under the scope of the Code and the Philippine Milk Code because not all promotional materials captured during the monitoring period were available for assessment by the authors. For online materials, the contents of some links were found to be broken and were not included in the assessment. Privacy restrictions prevented the collection of marketing materials on individual or private social media pages, and thus only public social media content was assessed. The monitoring did not include e-commerce sites and ad placements in online advertising platforms that display brief advertisements, service offerings, product listings, or videos to web users. Monitoring and assessment of ad placements in social media platforms and mobile apps were also not included in the scope of this study. For TV, the monitoring was limited to primetime, which was a total of 6 h per day. It also has to be noted that while the data coding was done by technical experts on the Code and the Philippine Milk Code, and the results were reviewed and cross-checked by four more technical experts, there was no reliability measurements performed to check the stability, reproducibility, and accuracy of the coding results.

## Conclusion

Our media monitoring and content analysis show evidence of circumventions of the Philippine Milk Code in modern and traditional media channels that exploit gaps in the legislation and its enforcement. The Philippine government, with support from development partners and breastfeeding advocacy coalitions should jointly enhance enforcement of the Philippine Milk Code and adopt other regulatory measures that provide safeguards against false and misleading health and nutrition claims, emotionally manipulative language in the promotion of products intended for use by children, and exploitative digital marketing strategies that circumvent marketing regulations. Regulatory agencies and civil society actors that support the Philippine Milk Code monitoring and enforcement in the Philippines should remain vigilant in monitoring promotion in traditional media channels and include regular media audits in routine monitoring.

## Data availability statement

The original contributions presented in this study are included in the article/Supplementary material, further inquiries can be directed to the corresponding author.

## Author contributions

PZ, JD-S, DC, and CM-S conceptualized the study, worked on the study design, data analysis, curation, and validation. DC, JD-S, CM-S, PZ, JC, TN, and RM contributed in drafting the manuscript, reviewing, and editing. All authors contributed to the article and approved the submitted version.

## Author disclaimer

The views and opinions set out in this article represent those of the authors, and do not necessarily represent the position of the foundation. Under the grant conditions of the Foundation, a Creative Commons Attribution 4.0 Generic License has already been assigned to the author Accepted Manuscript version that might arise from this submission.
