# Supplementary material for: Cross-sectional multimedia audit reveals a multinational commercial milk formula industry circumventing the Philippine Milk Code with misinformation, manipulation, and cross-promotion campaigns
Source: Front Nutr. 2023 Feb 2;10:1081499. doi: 10.3389/fnut.2023.1081499 (PMC9932888; doi:10.3389/fnut.2023.1081499)
Supplement: Supplementary file 1 [file Table_1.PDF]

Supplementary Table 1. List of keywords used in the monitoring of TV, print, and online materials

General keywords			
Breastfeed/Breast feed	Formula milk	Maternal milk	Powdered milk
Breastfeeding/Breastfeeding	Follow-up/Follow-on formula	Milk	Teats
Breastmilk/Breast milk	Growing-up milk	Milk drink	Toddler milk
Breastmilk Substitute/Breast milk substitute	Infant milk/Infant formula	Milk supplement	
Bottles (and "baby"/"infant")	Lactating mothers	Milk for pregnant women	
	Malnutrition	Mother's milk	
**Manufacturer/brand/product specific keywords**			
1st choice	Hipp Organic Follow-on Formula	Nido Junior Organic	Similac for Diarrhea
Abbott	Hipp Organic Infant Formula	Nido Protectus	Similac For Spit Up Non-GMO
Alacta	Hipp Organic Kindermilk	Nuby	Similac For Supplementation
Alactagrow	Hipp Organic Milk Supplement	Nuk	Similac Gain Plus
Alactamil	Hipp Organic Mixed Vegetable Medley	Nutramigen LGG	Similac Gain School
Alaska Milk	Hipp Organic Sweet Squash and Chicken	Nutren Junior	Similac Gain Two
Anmum Concentrate	Isomil	Pediasure	Similac Neosure
Anmum Materna	Isomil Two	Pediasure Enteral 1.0 cal	Similac One
Appeton Weight Gain Junior	Jersey Milk	Pediasure Enteral 1.0 cal with fiber	Similac Organic
Aprica	Kalbe	Pediasure Enteral 1.5 cal	Similac PM 60/40
Aqiva	Lactum	Pediasure Enteral 1.5 cal with fiber	Similac Pro Total Comfort
Avent	Lactum 3+	Pediasure Grow and Gain	Similac Pro-advance
Beaba	Lactum 6+	Pediasure Grow and Gain with fiber	Similac Pro-sensitive
Bear Brand	Lansinoh	Pediasure Peptide 1.0 cal	Similac Sensitive
Bear Brand ChocoMilk	Mam	Pediasure Peptide 1.5 cal	Similac Sensitive 20
Bear Brand Jr.	Mead Johnson	Pediasure Plus	Similac Sensitive Non-GMO
Bear Brand Real Strawberry	Medela	Petite Creations	Similac Soy Isomil
Birch Tree	Milk Magic	Philips Avent	Similac Soy Isomil 20
Bonakid	Mimijumi	Pigeon/Pigeon baby bottle	Similac Special Care 20
Bonakid Pre-school 3+	Mom & Me	Playtex Baby	Similac Spit Up
Bonamil	Mom&baby	Pre Nan	Similac Tummi Care
Bonna	Morinaga BMT Soya	Prenagen Lactamom	Similac Tummi Care Two
Cerelac	Morinaga BMT-HP	Prenagen Mommy Emesis	Sustagen
Cerelac NutriPuffs	Morinaga Chil Mil Soya	Progress Pre-school Gold	Sustagen Junior 1+
Chicco/Chicco baby feeding bottle	Morinaga Chil Mil-HP	Promama	Sustagen Kid 3+
Choco Milk	Morinaga Chil School Soya	Promil Aqiva	Sustagen School 6+
Comotomo	Morinaga NL 33 Non Lactose	Promil Four	Thinkbaby
Dr. Brown's Baby/Dr. Brown Baby	Nan Al 110 Lactose Free	Promil Gold Four	Tommee Tippee
Enfagrow A+ four	Nan Optipro HW Four	Promil Kid	Wyeth
Enfagrow A+ three	Nan Optipro HW One	Promil LF	
Enfakid	Nan Optipro HW Three	Promil Organic	
Enfalac A+1	Nan Optipro HW Two	Promil Pre-school	
Enfalac A+2	Nan Optipro One	Pure Bliss by Similac	
Enfamama	Nan Optipro Three	S-26 Comfort Gold	
Enfamil A+ Catch up	Nan Optipro Two	S-26 Gold One	
Enfamil A+ Gentlease	Nan Sensitive	S-26 Gold Two	
Enfamil A+ One	Nankid	S-26 HA Gold	
Enfamil A+ Two	Nankid Optipro Four	S-26 LF Gold	
Enfant	Nestle	S-26 One	
Enfapro	Nestogen	S-26 Organic	
Farlin/Farlin baby mik bottle	Nestogen Four	S-26 Promil Gold	
Fonterra	Nestogen One	S-26 Promil Gold Three	
FrieslandCampina	Nestogen Three	S-26 Promil Organic	
Friso Three	Nestogen Two	S-26 Promil Three	
Friso Tour	Nestokid	Similac Advance	
Frisolac One	Nestokid Four	Similac Advance 20	
Frisolac Two	Nido Advanced Protectus 3+	Similac Advance Non-GMO	
Frisomum	Nido Advanced Protectus 5+	Similac Alimentum	
Gerber	Nido Advanced Protectus Junior	Similac Breastfeeding Supplement	
Hipp Organic	Nido Fortified		
Hipp Organic Beef and Vegetable Medley	Nido Junior		
