# Supplementary material for: Cross-sectional multimedia audit reveals a multinational commercial milk formula industry circumventing the Philippine Milk Code with misinformation, manipulation, and cross-promotion campaigns
Source: Front Nutr. 2023 Feb 2;10:1081499. doi: 10.3389/fnut.2023.1081499 (PMC9932888; doi:10.3389/fnut.2023.1081499)
Supplement: Supplementary file 2 [file Table_2.docx]

| Advertorial | For the purposes of this study, advertorial refers to media material combining information with product or brand promotion which has disclosures of being a paid or sponsored content. |
| --- | --- |
| Breastmilk substitute (BMS) | For the purposes of this study, BMS is defined as “any type of milk, in either liquid or powdered form, including soy milk and follow-up formula, that are specifically marketed for feeding infants and young children up to the age of 3 years” (definition in Republic Act 11148)^1^. It includes CMF for children <36 months old (CMF for children less than 6 months old, CMF for children 6-11 months old, and CMF for children 12-35 months old). BMS is also defined under Section 4(a) of the Philippine Milk Code as “any food being marketed or otherwise represented as a partial or total replacement for breastmilk whether or not suitable for that purpose”^2^. The Supreme Court Decision on G.R. 173034 interpreted that, with the lack of reference to a particular age-group of children, the Philippine Milk Code intends to protect and promote nourishment of children more than 12 months old^3^. |
| BMS feeding related accessories | For the purposes of this study, BMS feeding related accessories refer to feeding bottles and teats and pacifiers. Feeding bottles and teats are included among the products covered by the Milk Code. |
| Commercially available complementary food (CACF) | Refers to complementary food that are commercially processed and available in the market. Complementary foods are defined as solid, semi-solid and soft foods (both locally prepared and commercially manufactured) provided to children between the ages of 6 and 23 months to complement breastmilk^4^. |
| Cross-promotion | A form of marketing promotion where customers of one product or service are targeted with promotion of a related product^5^. This can include packaging, branding and labelling of a product to closely resemble that of another (brand extension), it can also refer to use of particular promotional activities for one product and/or promotion of that product in particular settings to promote another product^5^. |
| Commercial milk formula (CMF) for children 0-6 months old | Refers to commercially available formula milk specifically marketed for children 0-6 months old. Also commonly known as infant formula. |
| Commercial milk formula (CMF) for children 6-12 months old | Refers to commercially available formula milk specifically marketed for children 6-12 months old. Also commonly known as follow-up formula. |
| Commercial milk formula (CMF) for children 12-35 months old | Refers to commercially available formula milk specifically marketed for children 12 to below 36 months old. Also commonly known as growing-up milk. |
| Commercial milk formula (CMF) for children ≥36 months old | Refers to commercially available formula milk specifically marketed for children 36 months old and above. |
| Commercial milk formula for Pregnant Women (CMF-PW) | CMF-PW refers to formula milk products marketed as nutritional supplements for pregnant and lactating women. |
| Commercial milk formula (CMF) | Refers to commercially available formula milk. It includes artificial ultra-processed products for infants made from a variety of products, including animal milks, soybean and vegetable oils and promoted as part of a line of products that includes infant formula, follow-on formula, and/or toddler/growing-up products, and may include formula products for special medical purposes^6^. It also includes formula milk products marketed for older children and adults. |
| Health claims | Any representation that states, suggests, or implies that a relationship exists between a food or a constituent of that food and health^7^. A health claim includes—but is not limited—to the following: 1) a nutrient function claim that describes the physiological role of the nutrient in growth, development, and normal functions of the body; 2) any other function claim concerning specific beneficial effects of the consumption of foods; and 3) a reduction of disease risk claim relating to the consumption of a food or food constituent, in the context of the total diet, to the reduced risk of developing a disease or health-related condition^7^. |
| Marketing | Refers to promotion, distribution, selling, advertising, public relations, information services, internet promotion and communication and information dissemination, in whatever form including but not necessarily limited to mail, email, text messages, telephone calls, website advertising, television, motion pictures, stage plays and radio programs whether live or taped^8^. For the purposes of this study, all unique marketing materials were included in the analysis even if not evident that they were paid for to promote or advertise the product. |
| Milk product  and other beverages marketed with no specific age range indicated | For the purposes of this study, “milk product and other beverages marketed with no specific age range indicated” refers to commercially available milk (in liquid or powder form, flavored or unflavored) and other popular commercially available beverages commonly given by parents to children that are marketed with no clearly defined age. Products specifically marketed as adult milk are not included under this classification. |
| Nutrition claims | Any representation which states, suggests, or implies that a food has particular nutritional properties including but not limited to the energy value and to the content of protein, fat, and carbohydrates, as well as the content of vitamins and minerals^7^. |
| Promotion | Employing any method, scheme, or design of directly or indirectly encouraging or enticing people or groups of persons in whatever form, whether by chance or skill to purchase or acquire products within the scope of the Milk Code^8^. |
| **References**  **^1^** Republic of the Philippines. Republic Act 11148 - An Act Scaling Up the National and Local Health and Nutrition Programs Through a Strengthened Integrated Strategy for Maternal, Neonatal, Child Health and Nutrition in the First One Thousand (1000) Days of Life, Appropriating Funds Therefor and for Other Purposes (2018). Available online: https://www.officialgazette.gov.ph/downloads/2018/11nov/20181129-RA-11148-RRD.pdf (accessed on 2 July 2022).  **^2^** Republic of the Philippines. Executive Order No. 51: National Code of Marketing of Breastmilk Substitutes, Breastmilk Supplement and Other Related Products (1986). Available online: <https://www.fda.gov.ph/wp-content/uploads/2021/05/Executive-Order-51.pdf> (accessed on 2 July 2022).  **^3^** Supreme Court En Banc, Republic of the Philippines. G.R. 173034 – Pharmaceutical and Health Care Association of the Philippines, petitioner, vs. Health Secretary Francisco T. Duque III; Health Under Secretaries Dr. Ethelyn P. Nieto, Dr. Margarita M. Galon, Atty. Alexander A. Padilla & Dr. Jade F. del Mundo; and Assistant Secretaries Dr. Mario C. Villaverde, DR. David J. Lozada and Dr. Nemesio T. Gako, respondents (October 2007). Available online: <https://elibrary.judiciary.gov.ph/thebookshelf/showdocs/1/44615> (accessed on 2 July 2022).  ^4^ United Nations Children’s Fund (UNICEF). Improving Young Children’s Diets During the Complementary Feeding Period: UNICEF Programming Guidance; 2020. Available online: <https://www.unicef.org/media/93981/file/Complementary-Feeding-Guidance-2020.pdf> (accessed 2 July 2022).  **^5^** World Health Organization (WHO). Guidance on ending the inappropriate promotion of foods for infants and young children: implementation manual. WHO: Geneva, 2017; ISBN 978-92-4-151347-0. Available online: <https://apps.who.int/iris/bitstream/handle/10665/260137/9789241513470-eng.pdf> (accessed on 2 July 2022).  ^6^ World Health Organization (WHO) & United Nations Children’s Fund (UNICEF). How the marketing of formula milk influences our decisions on infant feeding. WHO and UNICEF: Geneva, Switzerland, 2022; p.ix, ISBN 978-92-4-004460-9. Available online: <https://www.who.int/publications/i/item/9789240044609> (accessed on 2 July 2022).  ^7^ Food and Agriculture Organization (FAO). Guidelines for Use of Nutrition and Health Claims (CAC/GL 23-1997); adopted in 1997, revised in 2004, amended in 2001, 2008, 2009, 2010, 2011, 2012 and 2013 with Annex adopted 2009. Available at <http://www.fao.org/ag/humannutrition/32444-09f5545b8abe9a0c3baf01a4502ac36e4.pdf>. (accessed 2 July 2022).  ^8^ Department of Health, Republic of the Philippines. Revised Implementing Rules and Regulations of Executive Order No. 51. Otherwise Known as the “Milk Code”, Relevant International Agreements, Penalizing Violations Therof, and for Other Purposes (15 May 2006). Available online: <https://www.fda.gov.ph/wp-content/uploads/2021/05/Administrative-Order-No.-2006-0012.pdf> (accessed 2 July 2022). | |
