# Supplementary figures and images for: Cross-sectional multimedia audit reveals a multinational commercial milk formula industry circumventing the Philippine Milk Code with misinformation, manipulation, and cross-promotion campaigns
Source: Front Nutr. 2023 Feb 2;10:1081499. doi: 10.3389/fnut.2023.1081499 (PMC9932888; doi:10.3389/fnut.2023.1081499)

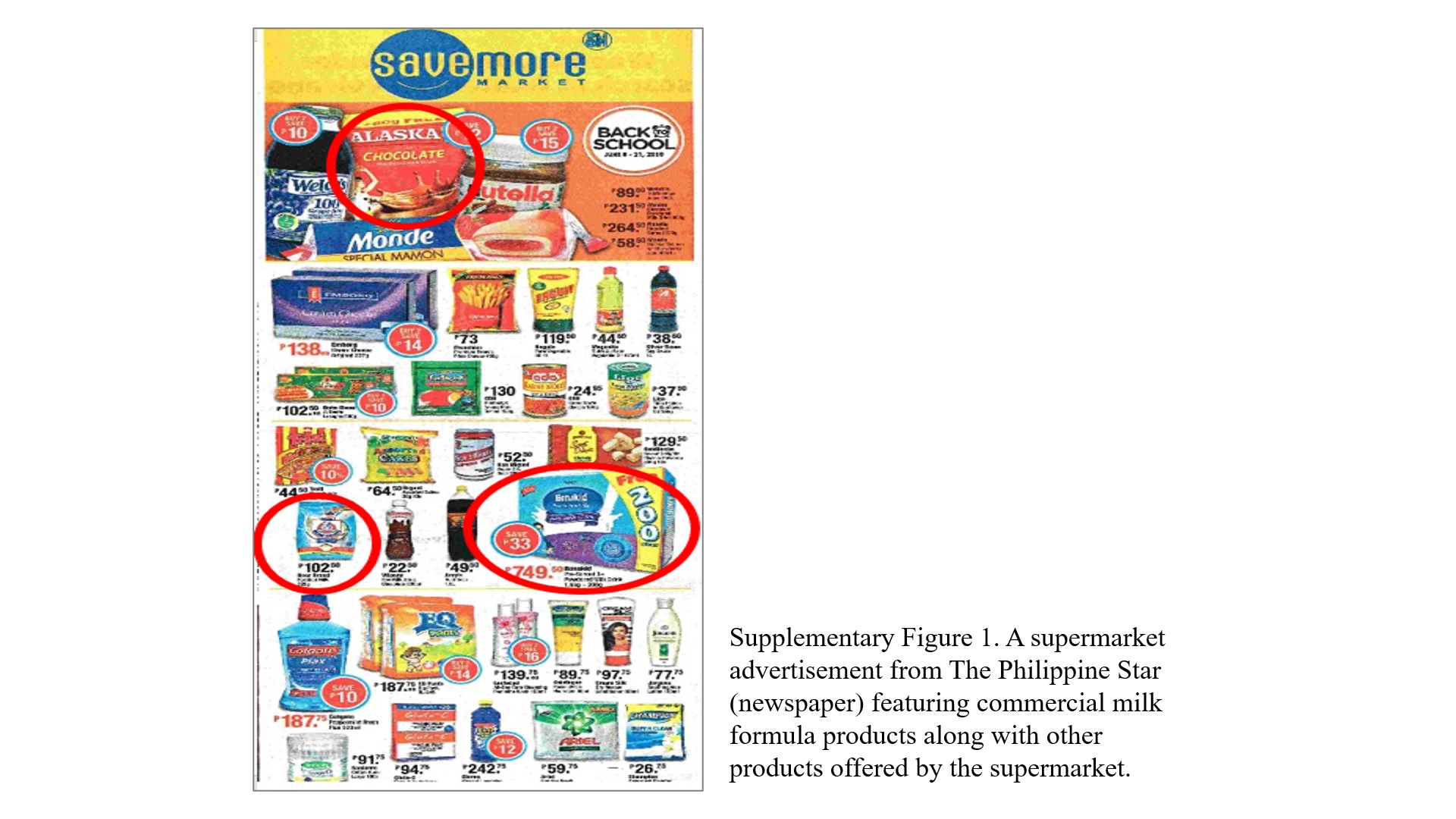

Supplement: Supplementary file 3 [file Image_1.jpg]

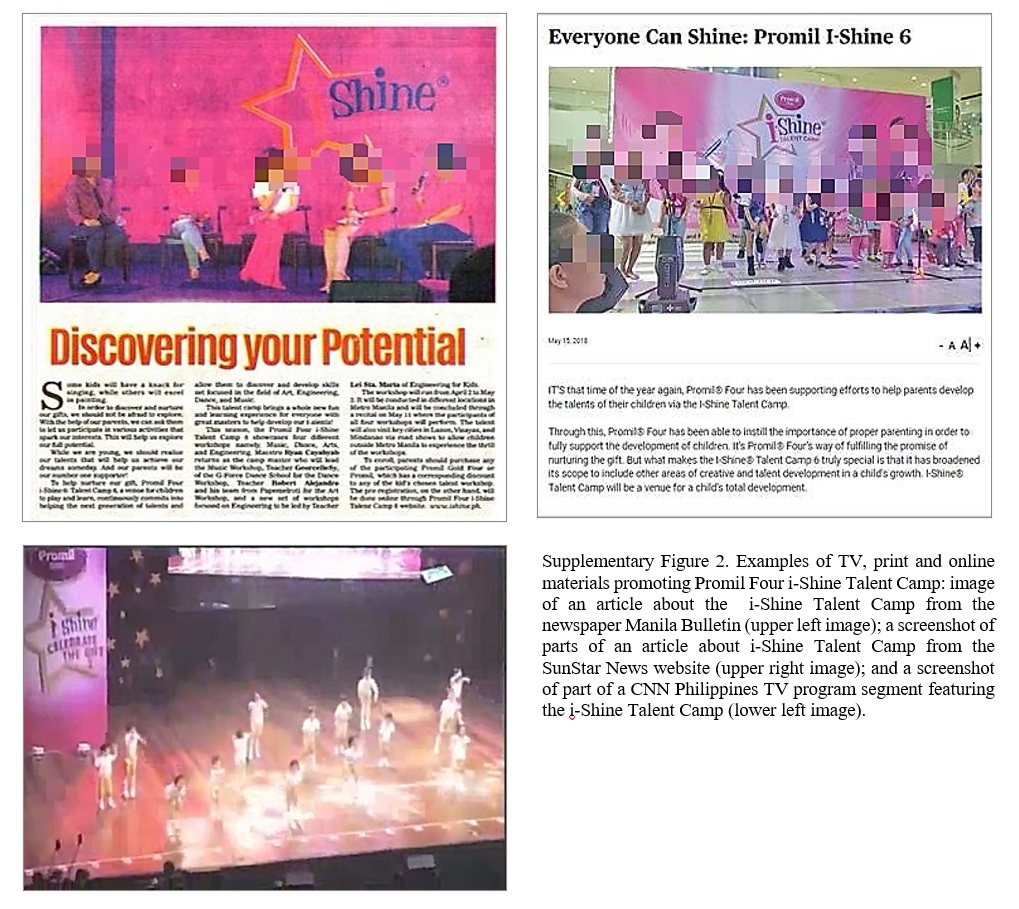

Supplement: Supplementary file 4 [file Image_2.jpg]
